# A biphasic response to blueberry supplementation on depressive symptoms in emerging adults: a double-blind randomized controlled trial

**DOI:** 10.1007/s00394-023-03311-9

**Published:** 2024-02-01

**Authors:** Martin Velichkov, Zsofia Bezur, Carien M. van Reekum, Claire M. Williams

**Affiliations:** School of Psychology and Clinical Language Sciences, University of Reading, Reading, UK

**Keywords:** Blueberries, Anthocyanins, Depression, Executive function, Randomized controlled trial

## Abstract

**Purpose:**

The aim of the present study was to examine the acute and chronic effects of wild blueberry supplementation on mood, executive function, and serum biomarkers of neuroplasticity, inflammation, and oxidative stress in emerging adults with moderate-to-severe depressive symptoms.

**Methods:**

In this double-blind trial, 60 emerging adults (*M*_age_ = 20.0 years, 32% male) with self-reported depressive symptoms were randomly assigned to receive a single blueberry drink (acute phase), followed by 6 weeks of daily blueberry supplementation (chronic phase), or a matched placebo drink. The primary outcome was Beck Depression Inventory-II (BDI-II) scores at 6-week follow-up. Further measures included momentary affect (PANAS-X) and accuracy on an executive function task. The data were analyzed using ANCOVAs adjusted for baseline values, sex, and habitual fruit and vegetable intake. Estimated marginal means were calculated to compare the treatment arms.

**Results:**

The blueberry drink significantly improved positive affect (*p* = 0.026) and executive function (*p* = 0.025) at 2 h post-ingestion, with change scores being positively correlated in the blueberry group (*r* = 0.424, *p* = 0.017). However, after six weeks of supplementation the reduction in BDI-II scores was greater in the placebo group by 5.8 points (95% CI: 0.8–10.7, *p* = 0.023). Generalized anxiety and anhedonia also decreased significantly more in the placebo group. No significant differences were found for any of the biomarkers.

**Conclusions:**

Six weeks of wild blueberry supplementation were inferior to placebo in reducing depressive symptoms. Nevertheless, the correlated improvements in positive affect and executive function after a single dose of blueberries point to a beneficial, albeit transient, psychological effect. These contrasting results suggest a biphasic, hormetic-like response that warrants further investigation.

**Trial registration**: NCT04647019, dated 30 November, 2020.

**Supplementary Information:**

The online version contains supplementary material available at 10.1007/s00394-023-03311-9.

## Introduction

Depression encompasses a spectrum of chronic conditions characterized by prolonged periods of low mood and anhedonia—a loss of interest or pleasure in activities that were previously enjoyable. The most common types are major depressive disorder (MDD), persistent depressive disorder (also called dysthymia), and seasonal affective disorder [1]. These conditions impose huge costs to individuals and society in terms of disability and mortality, healthcare costs, and economic and psychosocial losses. More than 280 million people suffer from depressive disorders worldwide, which makes them the leading contributor to chronic disease burden [2]. In developed countries, the lifetime prevalence of MDD is estimated to be as high as 30.1% for women and 17.4% for men [3]. Depression is also a major risk factor for suicide with more than half of suicide deaths occurring during a depressive episode [4]. Finally, depressive disorders are associated with an increased risk of developing various health conditions such as dementia and cardiovascular disease, thereby further increasing the burden of disease [5].

Besides low mood and anhedonia, other key symptoms of depressive disorders include fatigue or low energy, disturbances in appetite and sleep, cognitive dysfunction, negative self-cognitions (e.g., feelings of worthlessness and guilt), and suicidal ideation [1]. On a biological level, depression is characterized by increased stress sensitivity, disrupted neuroplasticity, which manifests peripherally as abnormally low serum concentrations of brain-derived neurotrophic factor (BDNF), and elevated levels of pro-inflammatory and oxidative stress markers, all of which are thought to play a role in the pathophysiology of the disease [5]. Psychologically, individuals with depression tend to interpret emotionally ambiguous information (including social information such as facial expressions) more negatively compared to healthy controls [6]. This “negative interpretation bias” correlates with depression severity, thus serving as an indirect marker of depressive symptomatology [7]. The management of depression includes pharmacotherapy (typically, monoaminergic antidepressants), psychotherapy, or a combination of both. However, poor response rates limit both treatment modalities [8, 9]. Moreover, antidepressants can cause serious side effects and withdrawal symptoms [10], whereas psychotherapy remains difficult to access for most patients for reasons of availability, affordability, or other barriers to getting care. Thus, there is a clear need to explore novel therapeutic approaches to alleviate the burden of depression.

Accumulating evidence suggests that dietary interventions are safe and effective for the treatment of depression. Several recent randomized controlled trials (SMILES [11], HELFIMED [12], and AMMEND [13]) have demonstrated that a Mediterranean-style diet significantly improves mental health in individuals with clinical levels of depression. This dietary pattern emphasizes the consumption of plant-based whole foods (whole grains, fruit and vegetables, legumes, nuts, olive oil) while discouraging the consumption of ultra-processed foods and red meat [14]. Among individual diet components, fruit and vegetables (FV) may be particularly important for mental health due to their high content of fibre, micronutrients, and beneficial phytochemicals. In the HELFIMED trial, the strongest correlation between reduced depression and changes in diet was observed for increased vegetable diversity, whereas greater fruit diversity had the strongest correlation with improvements in the qualify-of-life domains “mental health” and “happiness” [12]. These results are in accordance with the substantial body of epidemiological evidence associating FV consumption with better mental health outcomes [15]. However, very few experimental studies have specifically examined the effects of FV interventions on mental health. Conner et al. (2017) showed that young adults with a low FV intake (< 3 servings/day) who were provided with two additional daily servings of fresh FV over a two-week period experienced improvements in several aspects of psychological well-being (vitality, flourishing, and motivation) compared to a control group, but no changes in depression or anxiety symptoms were observed [16]. Moreover, mildly hypertensive middle-aged adults who consumed a high-polyphenol diet (5 servings of FV, one serving of berries, and 50 g dark chocolate/day) for eight weeks had significant improvements in depressive symptoms and quality of life compared to a control group consuming no more than two servings of FV per day [17]. These findings are promising, but more research is needed to develop and assess FV interventions, and understand their mechanisms of action, particularly in populations at high risk of mental ill-health and poor nutrition.

Among fruits and vegetables, berries seem to hold the greatest potential for improving mental health due to their exceptionally high content of polyphenols, particularly anthocyanins. Not only do these substances exert anti-inflammatory [18] and anti-oxidative effects [19], but they may also regulate neurotransmitter metabolism and support neuroplasticity in ways that counteract the pathophysiological processes of depression [20]. In addition, berries and other high-flavonoid foods have been shown to improve measures of cognitive performance [21] (such as executive function, which is often impaired in depression) and increase peripheral levels of BDNF with chronic administration [22]. In a cross-sectional study of 16,925 US adults, berries had the strongest protective association with depression among 10 groups of fruit and vegetables that were examined. Individuals who consumed an average of at least 67 g berries a day were 52% less likely to report clinical levels of depression compared to non-consumers [23]. A couple of randomized controlled trials (RCTs) support the efficacy of blueberry interventions to reduce depressive symptoms [24, 25], and there is preliminary evidence that even a single administration of a blueberry drink can enhance positive affect [26]. However, these studies have been conducted in non-selected samples from the general population, so it remains unclear whether the benefits would extend to individuals with clinical levels of depression.

While depressive disorders affect people of all ages, there is a particularly high risk during emerging adulthood, a distinct developmental stage between the ages of 18 and 25 years [27]. This vulnerability can be explained by a combination of often-stressful life transitions, lack of appropriate coping skills, and continued neurodevelopmental changes [28]. At the same time, diet quality in emerging adulthood is typically worse compared to any other life stage [29], which might contribute to the high incidence of depressive disorders [30]. Importantly, young adults have the lowest fruit and vegetable intakes of all age groups [31, 32]. These factors highlight the importance of developing nutritional interventions that can prevent and/or treat mental health problems specifically in emerging adults.

Considering the burden of depression and the potential of berries to improve mental health, the primary purpose of this RCT was to examine the antidepressant effects of 6 weeks daily blueberry intervention in emerging adults with moderate-to-severe depression. The secondary objectives included an assessment of the acute effects (2 h post-ingestion) on mood and executive function as well as the chronic effects of blueberries on depression-related indices (e.g., anxiety, anhedonia, quality of life), social cognitive processing (as a marker of depression severity), and serum biomarkers of neuroplasticity, inflammation, and oxidative stress. We hypothesized that the intervention would improve mental health outcomes and increase peripheral concentrations of BDNF while decreasing inflammation and oxidative stress. Furthermore, we expected an improvement in executive function following acute and chronic blueberry administration and a reduction of the negative interpretation bias of social information following chronic supplementation.

## Methods

### Study design

This study employed a randomized, double-blind, placebo-controlled design with both acute (2 h) and chronic (6 weeks) evaluations of a wild blueberry intervention on depressive symptoms and other health-related indices. It was registered prospectively (NCT04647019) and approved by the University of Reading Research Ethics Committee (UREC 20/13). All participants gave written informed consent, and the trial was conducted in accordance with the ethical standards laid down in the 1964 Declaration of Helsinki and its later amendments. The compensation for completing the study was £120 or research participation credits.

### Participants

A mixed-design ANOVA carried out on data from a previous study in adolescents demonstrated a medium effect size (partial η^2^ = 0.04) of a four-week blueberry intervention on depressive symptoms [24]. An a priori analysis in G*Power with this effect size indicated that a sample of 60 participants would be necessary to achieve a power level of 0.80 at a significance level of 0.05. Emerging adults with self-reported symptoms of depression were recruited via departmental email lists, posters, and social media from the student community of the University of Reading, UK. The inclusion criteria comprised 18–24 years of age, presence of moderate-to-severe depressive symptoms, defined as a Patient Health Questionnaire-9 (PHQ-9) total score ≥ 10 and a score ≥ 2 on the sum of items 1 and 2 [33], and willingness to provide venous blood samples. The exclusion criteria were any medically significant conditions (e.g., diabetes or gastrointestinal disorders), use of medication (excluding hormonal contraception, asthma and seasonal allergy drugs), history of mental illness other than anxiety and unipolar depressive disorders, receiving psychotherapy or counselling, and allergy to blueberries or any other *Vaccinium* fruits.

### Procedures

To determine eligibility and allow prospective participants to get familiar with the testing protocol, we invited them to attend a screening visit prior to the commencement of formal data collection. Eligible participants were allocated in a 1:1 ratio to either the blueberry condition or the placebo condition. A researcher not involved in recruitment or data collection used blocked randomization with random permuted blocks of two and four individuals to form the allocation list for the two comparison groups. The allocation sequence was generated using the online randomization tool Sealed Envelope (v1.19.1) and was concealed to the researchers enrolling and assessing participants until all analyses were complete. The first testing day included the acute phase of the study, which also served as the baseline assessment for the chronic phase. Participants arrived at the laboratory after an overnight fast and were provided with standard breakfast of 40 g porridge oats and 12 g stevia-sweetened vegan protein powder. After a short digestion break, participants completed the baseline task battery and mood questionnaire and had a venous blood sample taken. Then, they received a drink prepared by mixing 250 ml water with 22 g freeze-dried wild lowbush blueberries (*Vaccinium angustifolium*) or a blueberry-flavoured placebo drink matched for carbohydrates and fibre (see [34] for detailed chemical composition of the placebo powder). The blueberry drink contained the equivalent of 1 cup or 150 g fresh fruit with 121 mg anthocyanins (mostly delphinidin) and 55 mg chlorogenic acid, as quantified by liquid chromatography–mass spectrometry (see Supplementary Materials for detailed analytical characterization). The intervention drinks were prepared by a researcher not involved in the conduct of the trial and were administered in opaque shaker bottles. After a period of 2 h, the task battery and mood questionnaire were repeated, and participants were provided with a supply of 41 sealed sachets containing 22 g blueberry or placebo powder, which they were instructed to mix with water and consume every morning for 6 weeks. Participants were asked to write down the time when they consumed the intervention every day on a calendar designed for that purpose. Compliance was ≥ 88% in all participants and there was no evidence of differences between the groups. For the duration of the study, the sachets were stored in a freezer except during transportation. The final testing day included the standard breakfast, computer tasks, and blood sample. Thus, participants consumed the last dose of the intervention about 24 h before the final testing session.

### Outcome measures

The primary outcome was severity of depressive symptoms following 6 weeks of blueberry supplementation as measured by the Beck Depression Inventory-II (BDI-II), a validated self-report measure on a continuous scale from 0 to 63 points that covers a wide range of affective, cognitive, and somatic symptoms of depression [35]. The secondary measures that were assessed both acutely and at 6-week follow-up were momentary affect (mood) and executive function. Mood was assessed with the Expanded Form of the Positive and Negative Affect Schedule (PANAS-X) [36], which requires participants to indicate to what extent they feel different affective states on a 5-point Likert scale ranging from “not at all” to “extremely”. The positive affect (PA) scale had 22 items (e.g., happy, strong, motivated) and ranged from 0 to 88. The negative affect (NA) scale had 25 items (e.g., afraid, sad, hostile) and ranged from 0 to 100, with greater scores indicating worse mood. The PA and NA scales had excellent internal consistency with Cronbach’s α coefficients of 0.920 and 0.933 and average corrected item–total correlations of 0.572 and 0.580, respectively. Fatigue (4 items, range: 0–16) and calmness (3 items, range: 0–12) were also reported as exploratory outcomes.

**Fig. 1:**
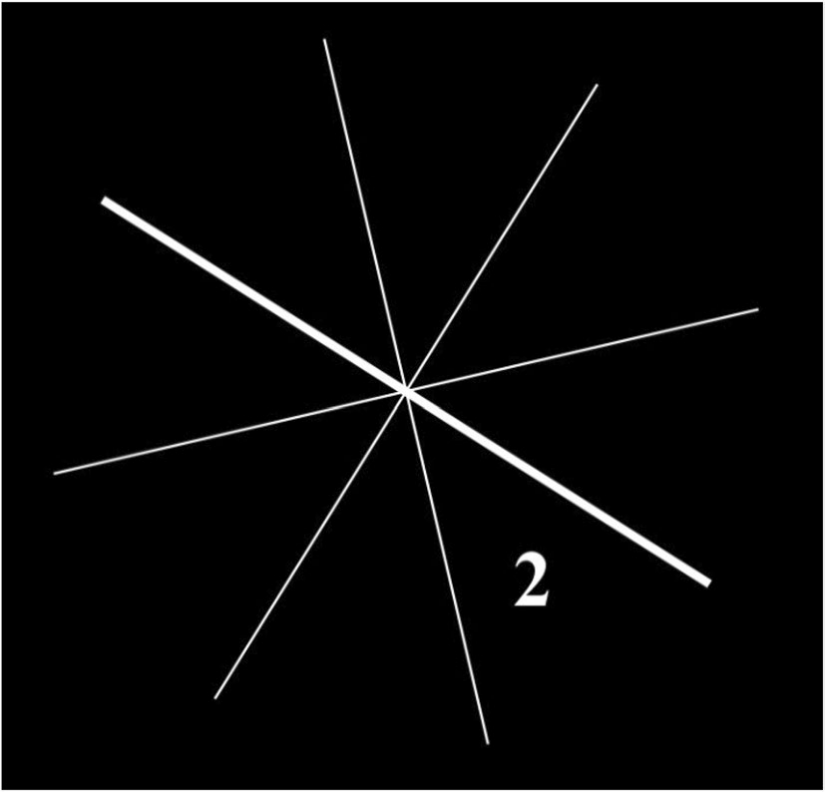
The task-switching test required participants to switch between two predictable tasks, which consisted of indicating whether random stimulus digits from 1 to 9 (excluding 5) displayed sequentially in a clockwise direction were odd or even (if shown above the bold line) or greater or less than 5 (if shown below the bold line)

Executive function was assessed with a modified version of a task-switching paradigm adapted by Miller et al. [37] to evaluate the performance costs that arise from switching between two predictable tasks. Participants viewed eight equally spaced segments of a circle divided in half by a bold white line (Fig. 1). A random stimulus digit from 1 to 9 (excluding 5) appeared sequentially in each segment in a clockwise direction. Each digit was displayed for a duration of 850 ms with an inter-stimulus interval of 500 ms. When the digit appeared in the segments above the bold line, participants had to indicate whether it was odd or even by pressing the relevant response key. When the digit appeared below the bold line, participants had to indicate if it was greater or less than five. Thus, the task switches every four trials, and the trial following the switch (switch trial) is considered the most cognitively demanding with higher error rates and response times [38]. During each session, participants completed eight practice rotations of the circle with feedback when a mistake was made, followed by 48 rotations (including 96 switch trials) without feedback. The primary outcome was the mean accuracy on switch trials expressed as percent correct, but we also reported the mean accuracy on the remaining 288 non-switch trials. Mean reaction times (RTs) were calculated separately for switch trials and non-switch trials, excluding values below 200 ms, which is considered the lower limit for a valid physiological response [39]. All participants completed a full practice session of the task during the screening visit to reduce the influence of practice effects.

Further secondary measures included the 9-item Patient Health Questionnaire (PHQ-9), which is based on the DSM-5 diagnostic criteria of major depressive disorder [33], the 7-item Generalized Anxiety Disorder Scale (GAD-7) [40], a modified version of the Snaith–Hamilton Pleasure Scale (SHAPS) with a total score ranging from 0 to 56 points and higher values corresponding to great levels of anhedonia [41], and the 10-item Perceived Stress Scale (PSS-10) [42]. Interpretation bias was assessed with an emotion categorization task based on research by Neta et al. [43]. Participants were presented with a series of facial emotional expressions (72 surprised faces, 36 happy faces, and 36 angry faces) in random order and were asked to rate the displayed emotion as positive or negative using a two-alternative forced-choice button response. Each stimulus was presented for 500 ms, followed by an inter-stimulus interval of 500 ms. The faces were equally split between males and females for each emotion and the response buttons were counterbalanced across participants. Since surprised expressions can be interpreted as either positive or negative, their rating offers a relevant model for examining interpretation bias. Thus, negative interpretation bias was operationalized as the percent of surprise trials rated as negative out of the total number of surprise trials excluding omissions. Accuracy on happy and angry faces was reported as a control measure.

For the assessment of biomarkers, venous blood samples were collected, left to coagulate for 30 min at room temperature, and centrifuged at 3000 rpm for 15 min to separate serum, which was then aliquoted and stored at − 80 °C until analysis. The serum concentrations of free BDNF and high-sensitivity interleukin-6 (IL-6) were assayed in duplicate with ELISA kits following the manufacturer’s protocols (Quantikine kit, R&D Systems Europe Ltd., Abingdon, UK). Samples below or above the detection limits of the IL-6 assay (*n* = 5) were imputed with the minimum or maximum value obtained. The concentrations of high-sensitivity C-reactive protein (hs-CRP) and uric acid were determined with a RX Daytona Plus automated clinical chemistry analyser (Randox Laboratories Ltd., County Antrim, UK). Individuals with hs-CRP levels > 10 mg/L (*n* = 5) were excluded from the analyses due to the likely presence of acute infection. Serum superoxide dismutase (SOD) was measured with a Randox reagent kit and expressed as units per litre (U/L) where one unit is the amount of SOD that inhibits the rate of formazan dye formation by 50%. As a further indicator of oxidative stress, we measured thiobarbituric acid reactive substances (TBARS) spectrophotometrically. Since 19.3% of the samples were below the detection limit, those values were imputed with the lower limit of detection for the assay (48 mmol/L). Finally, we attempted to measured oxygen radical antioxidant capacity (ORAC), but most of the samples were below the detection limits of the assay, so these results were not reported.

### Prognostic factors

A number of demographic, lifestyle and dietary variables relevant for the prognosis of depression were measured at baseline for the purpose of comparison between the two intervention arms. We assessed habitual diet using a modified version of the EPIC-Norfolk food frequency questionnaire [44], and reported daily energy intakes as well as the intakes of red and processed meat, fish and seafood, legumes and nuts, whole grains, fruit and vegetables, and berries. Hazardous alcohol use was measured with the 3-item Alcohol Use Disorders Identification Test (AUDIT-C) [45], and body mass index (BMI) was calculated by dividing weight (kg) by height squared (m^2^). In addition, we measured physical activity using the International Physical Activity Questionnaire–Short Form (IPAQ-SF) [46] and created four activity levels based on cut-off values from WHO guidelines [47]. Parental educational attainment was recorded as a proxy of socioeconomic status following the approach by Pascarella et al. [48]. A value of 0 was assigned if neither parent had post-secondary education, 1 if only one did, and 2 if both parents did.

### Statistical analyses

Descriptive statistics, including means with standard deviations (SD), medians for non-normally distributed variables, and frequencies for categorical variables, were reported for all prognostic factors and outcome measures. Baseline variables were compared between the two groups using independent samples *t*-tests, Mann–Whitney *U*-tests, and Chi-square tests, as appropriate. The effectiveness of the intervention was analysed using an intention-to-treat approach. In accordance with the statistical analysis plan, we carried out a series of ANCOVAs, in which post-intervention scores were the dependent variable, baseline scores were a covariate, and the independent variables were treatment (placebo vs. blueberry), sex (male vs. female), fruit and vegetable intake categorized as low (< 4 servings/day) vs. high (> 4 servings/day), and the interactions “treatment*sex” and “treatment*FV intake”. This cut-off of four servings was chosen to ensure feasibility, allowing for sufficient allocation of participants to both groups, while falling close to the national UK recommendation of consuming 5 portions of fruit and vegetables a day [31]. Estimated marginal means (EMMs) were calculated and compared using post-hoc pairwise LSD tests. To investigate any potential speed-accuracy trade-off and determine whether acute changes in mood were associated with acute changes in cognitive function, we calculated change scores by subtracting baseline values from the post-treatment values. Pearson's correlation analyses of these change scores were carried out separately in the two treatment groups and the corresponding correlation coefficients were compared using Fisher’s *r*-to-*Z*-transformation (two-tailed). Statistical outliers, defined as values beyond 1.5 interquartile ranges (IQR) from the first and third quartiles, were excluded from the respective analysis. In addition, we excluded participants with subclinical baseline symptoms, defined as scores < 13 on the BDI-II, < 9 on the PHQ-9, < 4 on the GAD-7, and < 8 on the SHAPS, from the respective chronic outcome analyses. The analyses were conducted with SPSS v28 (IBM Corp., Armonk, NY, USA) and statistical significance was defined as *p* < 0.05.

## Results

### Baseline measures and acute results

**Fig. 2:**
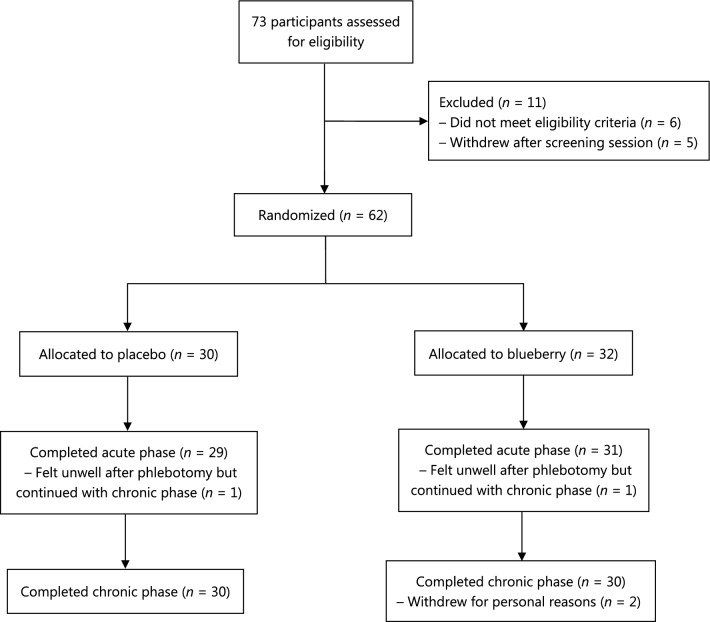
Participant flowchart. After screening, participants were randomly allocated to receive a single dose of either a blueberry drink or a placebo drink (acute phase) and were then provided with sachets of the respective intervention to consume at home for 6 weeks (chronic phase). The analyses followed an intention-to-treat approach

The participant flowchart is presented in Fig. 2 and the characteristics of participants who completed the chronic phase are shown in Table 1. There were no significant differences between the two intervention arms in terms of demographic, lifestyle or dietary factors. The duration of depressive symptoms was greater in the blueberry group (*Mdn* = 27 months) compared to the placebo group (*Mdn* = 19 months), but this difference fell short of statistical significance (Mann–Whitney *U* = 346, *p* = 0.122). Two-thirds of participants in the blueberry group had chronic depression, defined as an episode lasting ≥ 2 years [49], compared to half of the participants in the placebo group, *X*^2^ (1) = 1.71, *p* = 0.19. In addition, parental educational attainment was lower in the blueberry group (Mann–Whitney *U* = 564, *p* = 0.073), suggesting the blueberry group had a lower socioeconomic status. The baseline and post-intervention means and SD of the acute outcomes are displayed in Table 2. In each of the analyses for PA, NA and reaction time, there was one statistical outlier belonging to the placebo group. There were no significant baseline differences between the intervention arms for any of the measures (all *p*-values > 0.20). Accuracy and reaction times were inversely correlated, meaning that individuals with higher accuracy scores were also faster to respond. The correlations for switch trials and non-switch trials at baseline were *r*(60) = –0.631, *p* < 0.001 and *r*(60) = –0.437, *p* ≤  0.001, respectively.

**Table 1:** Demographic, lifestyle and dietary characteristics of participants by intervention arm

Characteristics	Placebo (*n* = 30)	Blueberry (*n* = 30)	*p-*Value
Age, years (SD)	20.1 (1.5)	19.9 (1.3)	0.65
Sex, % female	70	66.7	0.78
Duration of depressive episode, months (*Mdn*)	19	27	0.12
Ethnicity, % white	73.3	76.7	0.77
Low parental educational attainment^a^, %	26.7	46.7	0.07
Smoking, % occasional or daily	10	13.3	0.69
Hazardous alcohol use^b^, %	36.7	36.7	1.0
Vitamin D or multivitamin use, %	16.7	30	0.22
Hormonal contraception use, % (women only)	38.1	40	0.90
Vegetarian or vegan, %	10	13.3	0.69
Body mass index, kg/m^2^ (SD)	23.7 (4.4)	22.8 (2.5)	0.32
High physical activity^c^, % ≥ 35 MET-h/wk	36.7	30	0.71
Energy intake, kcal/d (SD)	1740 (638)	1723 (684)	0.92
Red and processed meat^d^, servings/wk	2.9	3.9	0.69
Fish and seafood^e^, servings/wk	0.9	1.2	0.60
Legumes and nuts^f^, servings/wk	2.1	2.0	0.82
Whole grains^g^, servings/wk	3.7	2.4	0.50
Fruit and vegetables^h^, servings/d	4.4	4.8	0.39
Berries^i^, servings/wk	0.9	0.9	0.79

Variable values are expressed frequencies (%), means and standard deviations (SD), or medians; * denotes *p* < 0.05

Unless otherwise indicated, *p*-values are based on chi-square tests for frequency variables, Mann–Whitney *U*-tests for dietary intakes and depression duration, and independent samples *t*-tests for age, BMI, and energy intake

^a^Defined as neither parent holding a university degree; *p*-value based on Mann–Whitney *U*-test with continuous scores

^b^Defined as an AUDIT-C score ≥ 8 for men and ≥ 7 for women

^c^*p*-value based on Mann–Whitney *U*-test with four activity categories

^d^Beef, beef burgers, pork, lamb, bacon, ham, luncheon meats, sausages, savoury pies

^e^Oily fish, white fish, fish fingers, shellfish

^f^Baked beans, pulses (e.g., lentils, beans, chickpeas), nuts and peanuts, peanut butter

^g^Wholemeal bread, crispbread, oat porridge, wholegrain cereals, wholewheat pasta, brown rice and quinoa

^h^Apples, pears, banana, grapes, citrus fruits, melon, peaches and plums, pineapple, mango, strawberries/raspberries, blueberries/blackberries, other fruit, carrots, broccoli, Brussels sprouts, green peas, green beans, courgette, cauliflower, parsnip/turnip, leek, onion, garlic, sweet peppers, green salad, spinach/kale (cooked), cucumber/celery, tomatoes, sweetcorn, beetroot, beansprouts, avocado, cabbage, mushrooms

^i^Strawberries/raspberries, blueberries/blackberries

**Table 2:** Unadjusted acute measures stratified by intervention arm

	Placebo (*n* = 29)				Blueberry (*n* = 31)				*p*-Value
	Baseline		2 h		Baseline		2 h		
	Mean	SD	Mean	SD	Mean	SD	Mean	SD	
Positive affect (0–88)	22.5	10.7	23.0	11.2	19.8	9.7	24.2	11.5	0.026*
Negative affect (0–100)	20.2	13.3	14.1	11.9	19.4	12.8	15.3	12.4	0.40
Calmness (0–12)	5.1	2.5	5.4	2.4	4.6	2.1	5.6	1.9	0.49
Fatigue (0–16)	7.9	4.6	7.0	3.8	8.6	3.7	7.4	3.7	0.41
Accuracy on switch trials, %	71.5	13.5	72.7	13.4	74.0	12.0	77.5	11.2	0.025*
Accuracy on non-switch trials, %	87.0	7.9	87.8	6.9	88.0	7.5	89.5	7.3	0.068†
Reaction time on switch trials, ms	580	49	555	49	564	52	556	59	0.033*
Reaction time on non-switch task, ms	526	41	504	41	515	52	506	53	0.050*

*p*-Values refer to the effect of treatment in ANCOVAs adjusted for baseline, sex, FV intake, and the interactions of treatment with sex and FV intake. One participant from the placebo group was excluded from the each of the analyses for PA, NA and reaction time due to being an outlier. † denotes *p* < 0.1 and * denotes *p* < 0.05

**Fig. 3:**
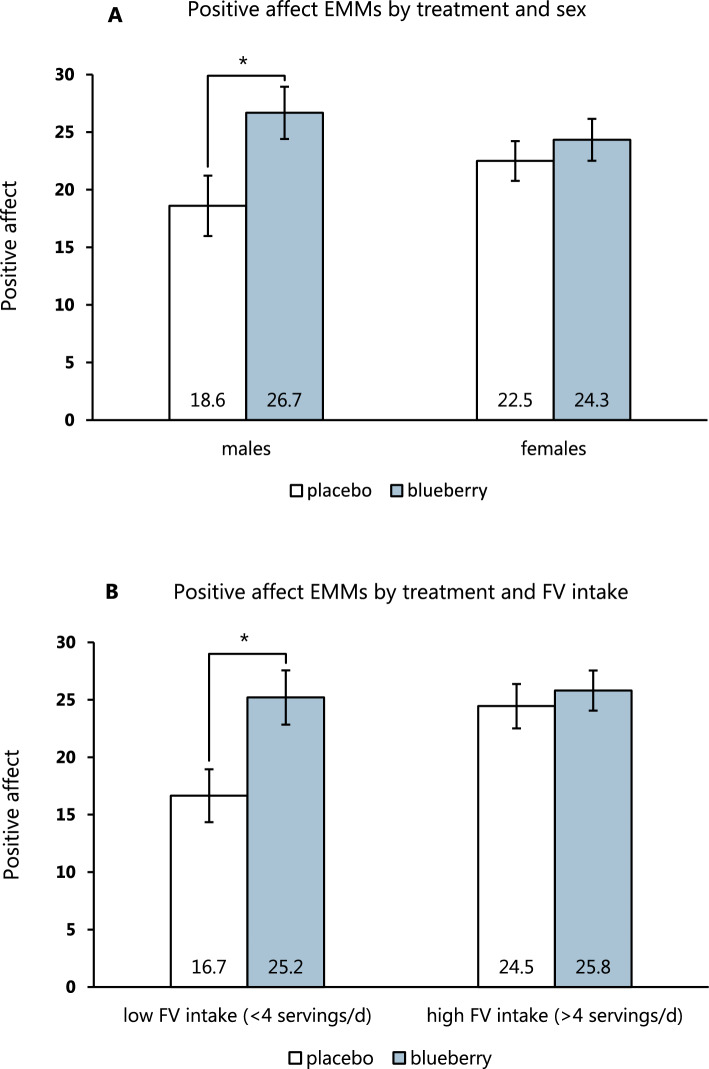
Baseline-adjusted means of positive affect (PA) stratified by treatment and sex (**A**) and treatment and fruit and vegetable (FV) intake (**B**). The pairwise tests showed significant differences between the placebo and blueberry groups in males (*p* = 0.026) and low FV consumers (*p* = 0.013). The values are evaluated at a baseline PA of 21.1 Error bars represent standard error of the mean. *EMM* estimated marginal means. * denotes *p* < 0.05

The ANCOVA analysis of PA revealed significant main effects of treatment: *F*(1,52) = 5.22, *p* = 0.026 and FV intake: *F*(1,52) = 4.18, *p* = 0.046. The baseline-adjusted mean PA of the placebo group was 20.6 (95% CI: 17.4–23.7) vs. 25.5 (95% CI: 22.5–28.5) in the blueberry group. In low FV consumers, baseline-adjusted PA was 20.9 (95% CI: 17.6–24.2) vs. 25.1 (95% CI: 22.5–27.7) in high FV consumers. There was no significant effect of sex: *F*(1,52) = 0.134, *p* = 0.72, but trends towards significance were found for the interactions between treatment and FV intake: *F*(1,52) = 3.06, *p* = 0.086 and treatment and sex *F*(1,52) = 2.11, *p* = 0.153. As shown in Fig. 3, the blueberry intervention improved PA to a greater extent in individuals with a low FV intake compared to those with high intake and was more effective in males than in females. Treatment had no significant effect on NA: *F*(1,52) = 0.73, *p* = 0.40 with a baseline-adjusted mean of 14.2 (95% CI: 11.3–17.2) in the placebo group and 16.0 (95% CI: 13.2–18.7) in the blueberry group. However, there was a borderline significant effect of sex on NA: *F*(1,52) = 3.99, *p* = 0.051 with a mean of 17.1 (95% CI: 13.9–20.3) in males and 13.1 (95% CI: 10.8–15.5) in females. None of the included variables had significant effects on calmness or fatigue.

**Fig. 4:**
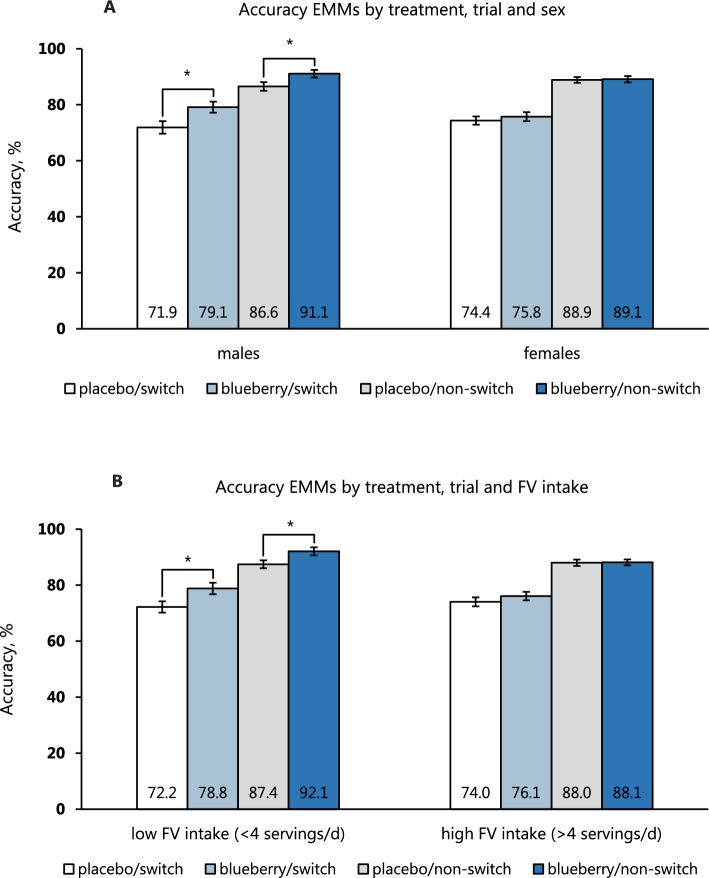
Baseline-adjusted mean accuracy stratified by treatment, trial difficulty, and sex (**A**) and fruit and vegetable (FV) intake (**B**). The pairwise tests showed significant differences between the placebo and blueberry groups in males on both switch trials (*p* = 0.018) and non-switch trials (*p* = 0.031). In addition, accuracy was greater in low FV consumers on switch trials (*p* = 0.028) as well as non-switch trials (*p* = 0.025). The values are evaluated at a baseline accuracy of 72.8 for switch trials and 87.5 for non-switch trials. Error bars represent standard error of the mean. *EMM* estimated marginal means * denotes *p* < 0.05

In terms of accuracy on switch trials, there was a significant effect of treatment: *F*(1,53) = 5.36, *p* = 0.025 with a baseline-adjusted mean of 73.1 (95% CI: 70.4–75.8) in the placebo group compared to 77.4 (95% CI: 74.9–80.0) in the blueberry group. The effect of treatment*sex on switch trial accuracy was *F*(1,53) = 2.60, *p* = 0.113 and that of treatment*FV intake: *F*(1,53) = 1.59, *p* = 0.212. A similar pattern was observed for accuracy on non-switch trials, although the effect of treatment fell short of statistical significance: *F*(1,53) = 3.47, *p* = 0.068. The baseline-adjusted mean accuracy on non-switch trials in the placebo group was 87.7 (95% CI: 85.9–89.6) vs. 90.1 (95% CI: 88.3–91.9) in the blueberry group. Trends towards significance were observed for the interactions treatment*sex: *F*(1,53) = 2.89, *p* = 0.095 and treatment*FV intake: *F*(1,53) = 3.23, *p* = 0.078. As shown in Fig. 4, the blueberry intervention increased accuracy to a greater extent in males compared to females and was also more effective in low FV consumers than in high FV consumers, with these differences being slightly greater on non-switch trials. Finally, the analysis of RT revealed a significant effect of treatment on switch trials: *F*(1,52) = 4.78, *p* = 0.033 and a borderline significant effect on non-switch trials: *F*(1,52) = 4.03, *p* = 0.050. The mean RT in the placebo group was 547 ms (95% CI: 535–558) on switch trials and 498 ms (95% CI: 489–508) on non-switch trials. In the blueberry group, these values were 564 ms (95% CI: 553–574) and 511 ms (95% CI: 502–520), respectively. There were no other significant effects (see Appendix for details).

**Fig. 5:**
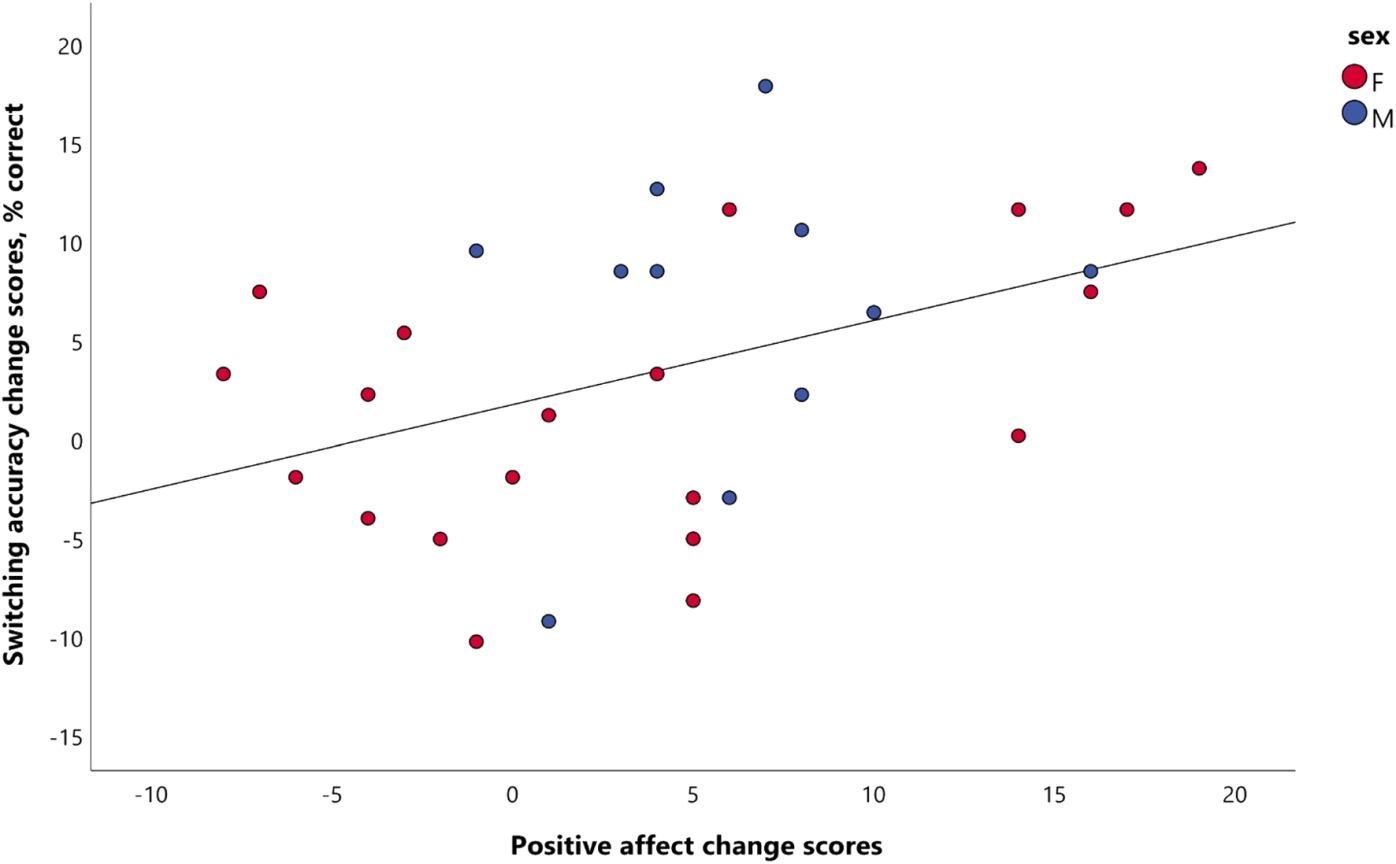
A scatterplot of positive affect change scores versus accuracy change scores on switch trials in participants who received the blueberry intervention (*n* = 31). Change scores were calculated by subtracting baseline values from the post-treatment values. A significant positive correlation was observed (*r* = 0.424, *p* = 0.017)

The correlations between change scores of positive affect, accuracy and reaction time for the two treatment groups are presented in Table 3. Changes in positive affect were significantly correlated with changes in switching accuracy in the blueberry group: *r*(29) = 0.424, *p* = 0.017 (Fig. 5), but not in the placebo group: *r*(26) = 0.312, *p* = 0.106. However, the two correlations were not significantly different (*z* = −0.47, *p* = 0.638) according to Fisher’s *z-*method. Accuracy change scores were not correlated with RT change scores in either group, indicating the absence of a speed–accuracy trade-off effect. None of the other correlations between changes scores were significantly different between the placebo group and the blueberry group.

**Table 3:** Correlations between acute change scores in the two treatment groups

	Placebo			Blueberry			*z*	*p-*value
	*r*	*n*	*p*-Value	*r*	*n*	*p-*Value		
ΔPA & ΔACC (switch)	0.312	28	0.106	0.424	31	0.017*	−0.47	0.638
ΔPA & ΔACC (non-switch)	0.306	28	0.113	0.148	31	0.427	0.61	0.542
ΔPA & ΔRT (switch)	−0.05	27	0.804	−0.107	31	0.565	0.21	0.834
ΔPA & ΔRT (non-switch)	0.087	27	0.667	0.073	31	0.698	0.05	0.960
ΔACC & ΔRT (switch)	0.214	28	0.275	−0.027	31	0.885	0.89	0.374
ΔACC & ΔRT (non-switch)	−0.207	28	0.290	0.162	31	0.384	−1.36	0.174

*p*-Values in the last column refer to the significance of the difference between two correlation coefficients using Fisher’s *r*-to-*Z*-transformations. One participant from the placebo group was excluded from the each of the analyses for PA and reaction time due to being an outlier

*ACC* accuracy; *PA *positive affect; *RT *reaction time

^*^denotes *p* < 0.05

### Chronic measures

**Fig. 6:**
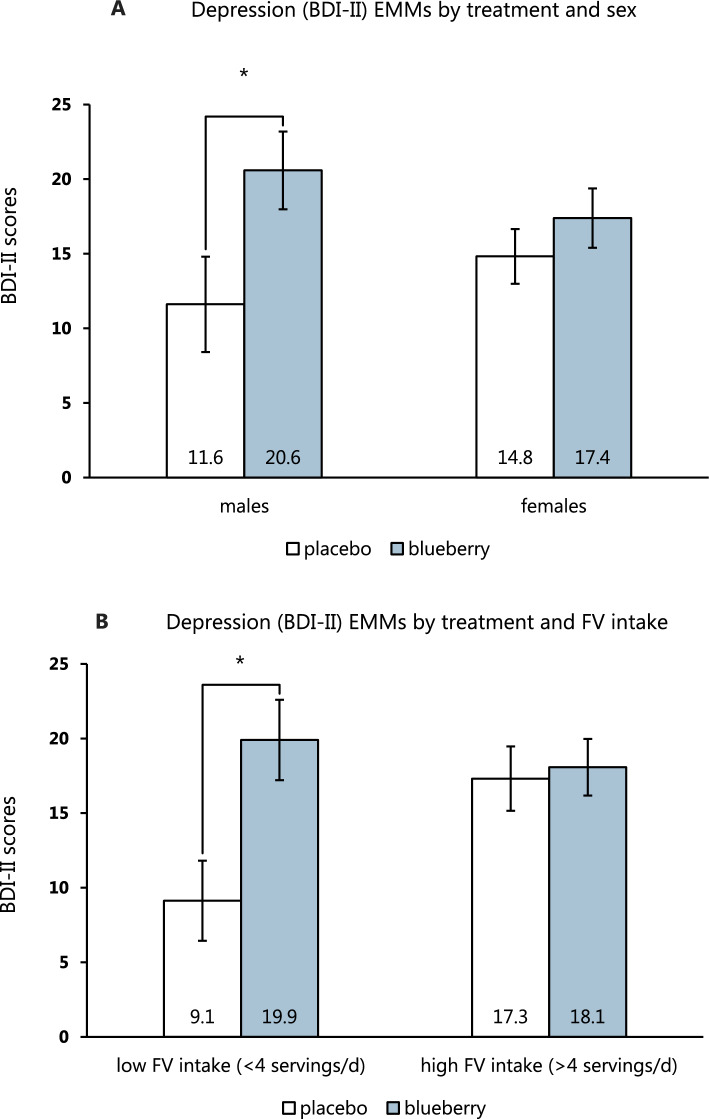
Baseline-adjusted means BDI-II scores stratified by treatment and sex (**A**) and treatment and fruit and vegetable (FV) intake (**B**). The pairwise tests showed significant differences between the placebo and blueberry groups in males (*p* = 0.031) and low FV consumers (*p* = 0.007). The values are evaluated at a baseline BDI-II score of 26.8. Error bars represent standard error of the mean. * denotes *p* < 0.05

The baseline and follow-up means and SD of the chronic outcomes are shown in Table 3. There were no significant baseline differences between the two groups for any of the measures (all *p*-values > 0.10). Three participants from the placebo group were excluded from the analysis for the primary outcome (two with BDI-II scores < 13 at baseline and one with a score greater than 1.5 times the IQR above the third quartile at follow-up). Thus, the range of BDI-II scores at baseline was from 13 to 48. After 6 weeks of treatment, BDI-II scores decreased significantly in both groups—12.1 points in the placebo group (95% CI: 8.0–16.3, *p* < 0.001) and 8.6 points in the blueberry group (95% CI: 5.2–11.9, *p* < 0.001). The ANCOVA showed a significant effect of treatment on depressive symptoms: *F*(1, 50) = 5.48, *p* = 0.023 with covariate-adjusted mean BDI-II of 13.2 (95% CI: 9.5–16.9) in the placebo group and 19.0 (95% CI: 15.6–22.3) in the blueberry group. There was also a significant treatment*FV intake interaction: *F*(1, 50) = 4.82, *p* = 0.033, which was driven by a greater reduction of depressive symptoms in the placebo group among low FV consumers compared to high consumers. BDI-II scores also decreased more in the placebo group among male participants compared to female participants although the interaction sex*treatment was not significant: *F*(1, 50) = 1.80, *p* = 0.187 (Fig. 6). The results for PHQ-9, generalized anxiety (GAD-7), and anhedonia (SHAPS) followed a similar pattern with significant effects of treatment favouring the placebo and interactions between FV intake and treatment on the PHQ-9 and SHAPS. There were no significant effects on perceived stress, momentary mood as measured by the PANAS-X, executive function or negative interpretation bias (see Appendix for detailed results).

The serum biomarker results showed no significant effect of treatment, but there was an interaction between treatment and FV intake on BDNF levels: *F*(1, 50) = 4.14, *p* = 0.047 as well as trends toward significance of treatment*FV intake on SOD levels: *F*(1, 50) = 4.02, *p* = 0.050 and TBARS: *F*(1, 47) = 2.87, *p* = 0.097 (Table 4). In low FV consumers, the blueberry treatment resulted in higher BDNF levels relative to placebo and lower levels of SOD and TBARS, while the reverse direction was observed among high FV consumers (see Appendix for details). However, these results should be interpreted with caution due to baseline differences between low and high FV consumers. As shown in Table 5, low FV consumers had significantly higher serum levels of BDNF and SOD but a lower level of uric acid compared to high FV consumers.

**Table 4:** Unadjusted chronic measurements stratified by intervention arm

	Placebo (*n* = 30)				Blueberry (*n* = 30)				*p*-Value
	Baseline		6 weeks		Baseline		6 weeks		
	Mean	SD	Mean	SD	Mean	SD	Mean	SD	
Beck Depression Inventory-II	27.0	9.1	14.9	9.0	26.6	8.3	18.0	9.0	0.023*
Patient Health Quiestionnaire-9	14.4	3.8	7.9	4.2	14.5	3.6	9.9	4.9	0.040*
Generalized anxiety (GAD-7)	10.1	4.2	5.4	2.9	10.5	3.9	7.5	3.8	0.026*
Anhedonia (SHAPS)	19.75	6.8	11.5	6.3	20.0	4.9	14.4	5.9	0.029*
Perceived stress (PSS-10)	27.0	5.2	21.6	6.5	26.1	4.1	22.0	6.4	0.49
Positive affect	22.3	10.9	28.6	10.2	19.9	10.4	29.4	11.1	0.90
Negative affect	20.4	13.1	13.8	13.2	19.1	13.1	15.7	13.9	0.33
Calmness	5.1	2.4	5.8	2.6	4.8	2.0	5.3	2.5	0.82
Fatigue	7.8	4.5	7.3	3.9	8.5	3.7	7.4	3.7	0.76
Accuracy on switch trials, %	71.4	13.4	74.0	13.4	73.9	12.2	76.2	10.9	0.99
Reaction time on switch trials, ms	584	51	575	54	562	52	570	53	0.11
Negative interpretation bias, %	72.0	15.3	65.7	19.2	71.1	13.9	66.7	16.7	0.70
Accuracy on happy faces, %	87.7	10.5	88.2	9.9	85.7	14.4	85.7	11.8	0.55
Accuracy on angry faces, %	93.8	5.4	91.1	8.2	92.6	9.4	91.6	7.2	0.41
BDNF, ng/mL	33.5	7.3	33.0	7.1	31.3	7.8	31.5	9.1	0.59
hs-CRP, mg/L	1.15	1.30	1.48	1.66	1.69	2.06	1.83	1.86	0.57
IL-6, pg/mL	0.98	0.71	1.15	1.15	0.94	0.61	1.13	0.68	0.68
Superoxide dismutase, U/L	352	149	347	188	319	109	313	49	0.30
TBARS, mmol/L	531	492	705	675	469	441	739	584	0.99
Uric acid, μmol/L	301	69	300	83	305	68	315	90	0.24

*p*-Values refer to the effect of treatment in ANCOVAs adjusted for baseline, sex, FV intake, and the interactions of treatment with sex and FV intake. Missing values and outliers are excluded from the analyses. *denotes *p* < 0.05

*BDNF* brain-derived neurotrophic factor; *hs-CRP *high-sensitivity C-reactive protein; *IL-6 *interleukin-6; *TBARS *thiobarbituric acid reactive substances

**Table 5:** Comparison of baseline serum biomarkers between low and high FV consumers

	Low FV consumers (*n* = 23)	High FV consumers (*n* = 39)	*p*-Value
BDI-II	25.9 (11.6)	26.7 (7.8)	0.75
Positive affect	20.0 (10.4)	22.6 (11.5)	0.37
BDNF, ng/mL	34.8 (8.9)	30.5 (6.0)	0.030*
hs-CRP, mg/L	1.46 (2.06)	1.48 (1.76)	0.97
IL-6, pg/mL	0.86 (0.62)	1.00 (0.67)	0.41
Superoxide dismutase, U/L	400 (178)	292 (50)	0.009*
TBARS, mmol/L	445 (464)	596 (496)	0.26
Uric acid, μmol/L	273 (61)	317 (69)	0.019*

*p*-Values are derived from independent samples *t*-tests. Missing values and outliers are excluded from the analyses. The values in the brackets are SDs

## Discussion

Depressive and anxiety disorders are a leading cause of global disease burden, particularly among young people. Recent research has highlighted the potential of nutritional interventions to improve mental health outcomes, but few studies have investigated the effects of single foods. Thus, the current study aimed to investigate the acute and chronic effects of wild blueberry supplementation on mental health and cognitive function in emerging adults with depressive symptoms. Our results showed a biphasic response to the blueberry intervention with acute improvements in mood and executive function but a lower recovery rate from depression following 6 weeks of daily supplementation compared to placebo. Importantly, these effects were stronger in individuals with a lower FV intake.

The acute improvements in mood and executive function are in agreement with previous research in healthy individuals. For example, a single blueberry drink was shown to significantly increase positive affect in both children and emerging adults [26]. Similarly, young adults who consumed a mixed-berry smoothie experienced an improvement in positive affect after 2 h, although this difference fell short of statistical significance when compared to the placebo group [38]. In addition, a number of studies have reported beneficial acute effects of berries on different domains of cognition, including memory and executive function [38, 50, 51]. Our finding that the blueberry drink caused a greater improvement in accuracy on cognitively demanding switch trials compared to non-switch trials is also consistent with previous findings in children [52]. Interestingly, we found a significant positive correlation between acute changes in momentary mood and changes in executive function in the blueberry group, but not in the placebo group. Thus, the positive correlation in the blueberry group suggests that the acute improvements in mood and cognition are driven by a common mechanism, such as modulation of cerebral blood flow or neurotransmitter activity. It has been demonstrated that a single administration of berries and other high-flavonoid foods (e.g., cocoa) increases flow-mediated dilation [53] and cerebral perfusion [54, 55], thus improving peripheral and central vascular function. This results in better oxygen and nutrient delivery to key brain areas, which can manifest psychologically as improved cognitive performance and elevated mood [56]. Moreover, berry flavonoids and their metabolites are capable of crossing the blood–brain barrier [57, 58] and modulating the metabolism of neurotransmitters within 1–2 h after ingestion. In particular, anthocyanins have been shown to inhibit the activity of monoamine oxidases (MAO)—the enzymes that break down serotonin, dopamine, and noradrenaline in the brain [59]. Several studies have replicated these effects in humans by demonstrating an inhibitory action of blackcurrant juice on MAO activity both peripherally and in the central nervous system (CNS) [51, 60]. Since monoamine neurotransmitters play a crucial role in the regulation of mood, alertness, motivation, and cognition [61, 62], increasing their CNS levels is expected to produce the kind of acute improvements that we observed in the blueberry group. On the other hand, the finding that reaction time decreased more in the placebo group at 2 h post-ingestion compared to the blueberry group is somewhat unexpected, as a meta-analysis indicates that blueberry interventions tend to decrease reaction time [63]. The fact that improvements in accuracy and reaction time were not correlated in the blueberry group rules out the possibility that this effect is due to a speed–accuracy trade-off (i.e., individuals taking longer to respond but achieving higher accuracy). A possible explanation is the difference in mean reaction times between placebo and blueberry at baseline (580 vs 564 ms), which could have given the placebo group greater room for improvement. Regardless, the baseline-adjusted post-treatment difference of 17 ms between the two groups is unlikely to be of clinical significance, considering that the improvement in accuracy was 4.3%.

In terms of the chronic outcomes, we observed a considerable reduction of depression symptoms in both treatment arms, this reduction being significantly greater in participants who received placebo than in those allocated to blueberry supplementation. The same pattern of results was found with respect to other depression-related mental health outcomes—diagnostic symptoms of depression (PHQ-9), generalized anxiety, and anhedonia. Several explanations might account for the considerable reduction of depression symptoms in both treatment arms. These include placebo response [64], spontaneous remission (including the impact of seasonal changes from winter to spring), regression toward the mean [65], the perceived demand characteristics of the study, and the Hawthorne effect, i.e., modifications of health behaviours in response to the awareness of being observed as part of a clinical trial [66]. For example, it is possible that participants improved their diet and physical activity, whether intentionally or not, which could have caused an improvement in mental health. While we assessed dietary intake and physical activity only at baseline, it would be worthwhile to repeat these assessments at follow-up in future studies.

The chronic results favouring the placebo are contrary to our hypothesis and to previous research suggesting that blueberry interventions might improve mental health. For example, Fisk et al. (2020) found that adolescents from the general population who consumed 13 g of freeze-dried blueberry powder for four weeks experienced a greater reduction in depressive symptoms compared to a placebo group [24]. Similarly, supplementation with blueberry juice for 20 days significantly reduced depression and anxiety symptoms in healthy adults while no changes were observed in the placebo group [25]. Previous cross-sectional studies have shown that individuals with higher intakes of berries or dietary anthocyanins are less likely to report symptoms of depression [23, 67, 68]. Nevertheless, two intervention studies in non-depressed older adults found no changes in depression scores following long-term blueberry supplementation, but reported significant improvements in cognitive function [37, 69]. In addition, neither berries nor dietary anthocyanins were associated with a lower risk of depression in a large cohort of women without a previous history of depression at baseline [70]. The present study differs from previous research in that it represents the first RCT to explore the antidepressant effects of blueberries in a sample with clinical levels of depression.

The initial improvement in positive affect followed by a relative deterioration of depressive symptoms is indicative of a biphasic, hormetic-like dose–response relationship. Hormesis is a phenomenon in which a chemical induces biologically opposite effects at different doses, most commonly a stimulatory effect at low doses and an inhibitory effect at high doses [71]. For example, quercetin, the most abundant flavonol in blueberries, has been described as an “in vivo antioxidant with strong hormetic potential” [72]. In particular, *C. elegans* nematodes grown in 5 μg/mL anthocyanin-rich bilberry extract showed improved resistance to thermal stress and lower accumulation of reactive oxygen species, but nematodes treated with 10 μg/mL extract had a higher mortality rate compared to controls, which was interpreted as a hormetic response [73]. On a cellular level, anthocyanins play an important role in the activation and release of nuclear factor erythroid 2 (Nrf2)—a key transcription factor that regulates the expression of a wide range of genes with cytoprotective functions [74]. It has been suggested that intermittent activation of Nrf2 produces beneficial physiological effects, whereas excessive, long-term stimulation may lead to detrimental effects [75]. No previous research has demonstrated a neurohormetic response associated with blueberry phytochemicals in humans, but there has been evidence that vitamin supplementation in certain populations might cause harmful health effects. In particular, smokers assigned to take β-carotene for a period of 5–8 years had a greater incidence of lung cancer and a higher mortality than individuals assigned to placebo [76]. Thus, the logic “if some is good, more is better” might not apply with regard to specific nutrients and patient populations. An important observation in the present study was that individuals with a lower FV intake (as well as male participants) were more responsive to both the beneficial acute effects of the blueberry intervention and to its detrimental chronic effects on depressive symptoms. It is possible that a high FV intake results in greater exposure to phytochemicals, which renders individuals less responsive to FV-based dietary interventions via habituation. Accordingly, previous studies that found positive changes on mood [16] or cognition [22] with a high-flavonoid diet recruited individuals with a low baseline FV intake. To maintain the acute improvements in mood in patients with depression and low FV consumption, an effective strategy could be to increase blueberry intake gradually to 2–3 servings a week and to incorporate them as part of a varied plant-centred diet rather than as monotherapy.

With regard to the changes in executive function following chronic (6 weeks) blueberry supplementation, our hypothesis that blueberry-treated individuals would improve more than the placebo group was not confirmed. Instead, the acute improvement in accuracy seen in the blueberry group was not maintained with chronic administration. It is possible that the acute cognitive enhancement was transient and repeated intake resulted in habituation. In comparison, a number of studies conducted in older adults have reported improvements in memory and/or executive function after long-term (12–24 weeks) interventions with blueberries [37, 69], strawberries [77], and cherries [78] (see [21] for a systematic review of RCTs). In line with this evidence, habitual intakes of berries and dietary anthocyanins have been associated with lower rates of cognitive decline in prospective cohorts of older adults [79, 80]. Hence, emerging adults, who are at the peak of their cognitive functioning age-wise, might be less susceptible to the effects of blueberries compared to older adults, whose cognitive capacities are in decline.

In addition to psychological outcomes, we studied the effects of chronic blueberry supplementation on serum biomarkers of neuroplasticity (BDNF), inflammation (hs-CRP and IL-6), and oxidative stress (SOD and TBARS), but found no differences between the two treatment arms at follow-up. In a previous study, 12 weeks of blueberry supplementation increased hippocampal BDNF levels in mice and improved spatial working memory [58]. Similarly, participants who consumed a high-flavonoid diet rich in berries experienced an increase in serum BDNF after 6 weeks, with levels continuing to rise up to 18 weeks [22]. The present study differs in several important ways. First, we administered a single food rather than providing participants with a range of different high-flavonoid fruits and vegetables. It is possible that consuming berries as part of a varied diet rich in FV is more beneficial than solely consuming blueberries without further diet changes. Second, participants in the previous study were much older (mean age of 51 years), had above-average risk of cardiovascular disease, and a low FV intake (< 4.4 portions/day). These factors could have made them more responsive to the high-flavonoid intervention. In terms of inflammation and oxidative stress, there is meta-analytic evidence that dietary anthocyanins can significantly reduce levels of CRP and IL-6 [18] while increasing SOD and total antioxidative capacity [19]. Overall, these effects tended to be stronger in individuals with impaired health (e.g., metabolic syndrome, diabetes) and in studies with a duration ≥ 8 weeks. Importantly, previous research has focused almost exclusively on middle-aged and older individuals, so little is known on how berries or anthocyanins affect inflammatory and oxidative stress markers in emerging adults. Finally, the finding that low FV consumers had a better biomarker profile characterized by higher serum levels of BDNF and SOD but a lower level of uric acid is unexpected. As mentioned above, intake of high-flavonoid FV increases serum BDNF [22], although a small observational study found lower BDNF levels among individual consuming ≥ 2 fruits a day [81]. In another study, FV consumption in men was positively associated with blood levels of SOD [82]. Thus, the present results warrant further investigation.

The principal strength of this study is its rigorous RCT design with both acute and chronic endpoints, combining a comprehensive set of self-reported measures, behavioural data, and biological markers. Some of the limitations include the short intervention length and the small sample, in which women were overrepresented. While some blueberry intervention studies have found effects on mood after only 3–4 weeks of supplementation [24, 25], it seems that longer periods are typically needed for detectable differences in cognition and biomarkers to appear. For instance, Siddarth et al. (2020) demonstrated that pomegranate juice improved visual memory performance in older adults after 12 months, but not 6 months, of supplementation [83]. Furthermore, our sample had a relatively high FV intake and high levels of physical activity compared to the national average for this age group [31], which can have implications for the generalizability of our findings. Likewise, our decision to recruit individuals with moderate-to-severe depressive symptoms was based on the rationale that this population stands to benefit most from the potential antidepressant effects of blueberries. However, it might be the case that berries are effective in the prevention of mental health disorders or during their subclinical stages (which would explain the protective effects in epidemiological studies) but perhaps counterproductive in the treatment of moderate to severe depression. Thus, in future studies, it might be worth focusing on individuals with mild/subclinical depression, as this population is also less likely to experience large changes in mood due to spontaneous remission or placebo response.

In conclusion, the present study found that 6-week placebo treatment outperformed wild blueberry supplementation in improving mental health indices in emerging adults with depressive symptoms. The intervention had no effect on serum biomarkers of neuroplasticity, inflammation or oxidative stress, and did not improve executive function. In contrast, the acute phase of the study showed that a single administration of blueberries improved positive affect and executive function, with those changes being positively correlated, which suggests the existence of a common mechanism, such as modulation of cerebral blood flow or neurotransmitter levels. Considering these conflicting results, further studies on the relationship between berry intake and mental health are warranted.

### Supplementary Information

Below is the link to the electronic supplementary material.

Supplementary file1 (PDF 101 KB)

## Data Availability

The data that support the findings of this study are available from the authors upon reasonable request.
