# Supplementary material for: A biphasic response to blueberry supplementation on depressive symptoms in emerging adults: a double-blind randomized controlled trial
Source: Eur J Nutr. 2024 Feb 1;63(4):1071–88. doi: 10.1007/s00394-023-03311-9 (PMC11139700; doi:10.1007/s00394-023-03311-9)
Supplement: Supplementary file 1 — Supplementary file1 (PDF 101 KB) [file 394_2023_3311_MOESM1_ESM.pdf]

Analytical characterization of freeze-dried blueberry powder

Characterization of freeze-dried blueberry powder obtained from Wild Blueberry Association of North America (WBANA) was carried out by Dr. Mario Ferruzzi, Professor in the Department of Food, Bioprocessing and Nutrition Sciences at the Plants for Human Health Institute, North Carolina State University. The composition of anthocyanins and other major constituents are listed in Table 1 and 2.

Table 1. Anthocyanins in freeze-dried blueberry powder as quantified by LC-MS method

	Anthocyanins in Freeze-dried Wild Blueberry Powder (mg/100 g powder)				
	Cyanidin	Peonidin	Petunidin	Malvidin	Delphinidin
Galactoside	20.08 ± 3.25	14.67± 0.84	20.35 ± 0.58	14.33 ± 0.27	127.01 ± 1.36
Glucoside	27.09 ± 1.57	10.66 ± 0.50	18.16 ± 0.37	20.55 ± 0.28	56.61 ± 2.08
Arabinoside	26.01 ± 2.45	11.56 ± 0.15	14.33 ± 0.27	16.72 ± 0.29	33.73 ± 0.70
Other	11.63 ± 0.08	9.46 ± 1.07	7.52 ± 0.04	9.59 ± 0.07	80.74 ± 1.26
Total	84.81	46.35	60.36	61.19	298.09

Table 2. Other major constituents in freeze-dried blueberry as quantified by LC-MS method

Other constituents in Freeze-dried Wild Blueberry Powder (mg/100 g powder)					
Chlorogenic Acid	Ferulic Acid	Epicatechin	Myricetin	Quercetin-3-Glucoside	Syringetin-3-Glucoside
248 ± 2.70	4.71 ± 0.11	2.87 ± 0.35	0.68 ± 0.04	31.19 ± 0.59	6.88 ± 0.34

MERIEUX NutriSciences

SILLIKER, Inc.
Illinois Laboratory
3600 Eagle Nest Drive, North Building, Crete, IL 60417
Tel. 877-777-6375 Fax. 312-729-1320

CERTIFICATE OF ANALYSIS

**COA No:**	CHG-41895675-0
**Supersedes:**	None
**COA Date**	12/18/18
**Page 1 of 1**	

TO:
Mr. Anandh Babu Pon Velayutham
Assistant Professor, Nutrition & Integra
University of Utah
250 South 1850 East #224C
Salt Lake City, UT 84117

**Received From:**	Salt Lake City, UT
**Received Date:**	12/7/18

Location of Test: (except where noted)
Crete, IL

Analytical Results

Desc. 1: FD-BB
Desc. 2: Freeze dired blueberry powder

Laboratory ID: 383435165
Condition Rec'd: NORMAL
Temp Rec'd (°C): 20

Analyte	Result	Units	Method Reference	Test Date	Loc.
Ash	1.94	% (w/w)	AOAC 945.46	12/13/18	
Calories by Calculation	406	Cal/100g	Atwater Factors	12/14/18	
Carbohydrates - Calculation	90.84	% (w/w)	Calculation	12/14/18	
Dietary Fiber			AOAC 991.43 (Mod.)	12/13/18	
TDF	15.64	% (w/w)			
Sample Modifications	None	-			
Fat - Mojo, Acid Hydrolysis	3.85	% (w/w)	AOAC 933.05	12/11/18	
Insoluble Dietary Fiber			AOAC 991.43	12/13/18	
Dried during analysis	No	-			
Defatted during analysis	No	-			
IDF as is	11.62	% (w/w)			
Moisture - Vacuum Oven	1.25	% (w/w)	AOAC 934.06	12/11/18	
Protein - Combustion			AOAC 992.23	12/13/18	
Protein Factor	6.25	-			
Protein	2.12	% (w/w)			
Soluble Dietary Fiber			AOAC 991.43	12/13/18	
Dried during analysis	No	-			
Defatted during analysis	No	-			
SDF as is	4.02	% (w/w)			
Sugars			AOAC 980.13	12/18/18	
Fructose	34.64	% (w/w)			
Glucose	33.48	% (w/w)			
Sucrose	<0.25	% (w/w)			
Maltose	<0.25	% (w/w)			
Lactose	<0.25	% (w/w)			

Jeff Wilson — Division Vice President

Results reported herein are provided "as is" and are based solely upon samples as provided by client. This report may not be distributed or reproduced except in full. Client shall not at any time misrepresent the content of this report. Mérieux NutriSciences assumes no responsibility, and client hereby waives all claims against Mérieux NutriSciences, for interpretation of such results.

Except as otherwise stated, Mérieux NutriSciences Terms and Conditions for Testing Services apply.
